# Distinct Signatures in the Receptor Repertoire Discriminate CD56bright and CD56dim Natural Killer Cells

**DOI:** 10.3389/fimmu.2020.568927

**Published:** 2020-12-01

**Authors:** Vera Schwane, Van Hung Huynh-Tran, Sarah Vollmers, Vivien Maria Yakup, Jürgen Sauter, Alexander H. Schmidt, Sven Peine, Marcus Altfeld, Laura Richert, Christian Körner

**Affiliations:** ^1^Research Department Virus Immunology, Heinrich Pette Institute, Leibniz Institute for Experimental Virology, Hamburg, Germany; ^2^Univ. Bordeaux, Inserm, Bordeaux Population Health Research Center, UMR1219 and Inria, team SISTM, Bordeaux, France; ^3^DKMS gemeinnützige GmbH, Tübingen, Germany; ^4^DKMS Life Science Lab, Dresden, Germany; ^5^Institute for Transfusion Medicine, University Medical Center Hamburg-Eppendorf, Hamburg, Germany; ^6^Department of Immunology, University Hospital Eppendorf (UKE), Hamburg, Germany

**Keywords:** NK cells, CD56bright, CD56dim, CD58, CD205, CD161, CD147, CD82

## Abstract

NK cells are phenotypically and functionally diverse lymphocytes due to variegated expression of a large array of receptors. NK-cell activity is tightly regulated through integration of receptor-derived inhibitory and activating signals. Thus, the receptor profile of each NK cell ultimately determines its ability to sense aberrant cells and subsequently mediate anti-viral or anti-tumor responses. However, an in-depth understanding of how different receptor repertoires enable distinct immune functions of NK cells is lacking. Therefore, we investigated the phenotypic diversity of primary human NK cells by performing extensive phenotypic characterization of 338 surface molecules using flow cytometry (n = 18). Our results showed that NK cells express at least 146 receptors on their surface. Of those, 136 (>90%) exhibited considerable inter-donor variability. Moreover, comparative analysis of CD56bright and CD56dim NK cells identified 70 molecules with differential expression between the two major NK-cell subsets and allowed discrimination of these subsets *via* unsupervised hierarchical clustering. These receptors were associated with a broad range of NK-cell functions and multiple molecules were not previously associated with predominant expression on either subset (e.g. CD82 and CD147). Altogether, our study contributes to an improved understanding of the phenotypic diversity of NK cells and its potential functional implications on a cellular and population level. While the identified distinct signatures in the receptor repertoires provide a molecular basis for the differential immune functions exerted by CD56bright and CD56dim NK cells, the observed inter-individual differences in the receptor repertoire of NK cells may contribute to a diverging ability to control certain diseases.

## Introduction

Natural killer (NK) cells are innate lymphocytes that mediate anti-viral (1) and anti-tumor (2) responses. NK-cell effector functions include direct lysis of target cells as well as production of pro-inflammatory cytokines such as IFNγ and TNFα (3). NK cells utilize a large array of germline-encoded surface receptors to interact with their environment, sense aberrant cells and subsequently mount potent effector responses. Among others, NK cells express receptors recognizing MHC class I molecules, stress ligands and cytokines as well as adhesion molecules and costimulatory receptors (4, 5). NK-cell activation is dependent on the integration of activating and inhibitory signals from multiple receptors. NK cells exert effector functions when signaling from their activating and costimulatory receptors outweighs signaling from their inhibitory receptors (4). Therefore, the receptor repertoire of a given NK cell ultimately determines its ability to sense and counteract environmental threats.

In humans, two major NK-cell subsets can be distinguished and are characterized by the differential expression of the adhesion molecule CD56 and the low-affinity Fc receptor CD16 (FcγRIIIa) (6, 7). They are commonly referred to as CD56bright and CD56dim NK cells. CD56dim NK cells predominate in peripheral blood whereas CD56bright NK cells constitute the majority of NK cells in secondary lymphoid tissues (e.g. lymph nodes) and several organ tissues (e.g. liver, uterus, and kidneys) (8). According to current understanding of NK-cell development, CD56bright and CD56dim NK cells both represent mature NK-cell subsets with distinct functional and phenotypic characteristics. Yet, CD56dim NK cells are more terminally differentiated and can arise from CD56bright NK cells (9–11). In the classical dichotomy, CD56dim NK cells are the cytotoxic “effector” NK cells while CD56bright NK cells are more “immunoregulatory,” i.e. produce vast amounts of cytokines and have superior proliferative capacities (3). CD56dim NK cells have higher expression of CD16, perforin and granzymes which enable them to mediate potent cytotoxicity and ADCC (8). However, this classical assignment of roles has been challenged by data showing enhanced cytotoxic responses from CD56bright NK cells upon stimulation with cytokines as well as vast production of cytokines by CD56dim NK cells following target cell recognition *via* surface-bound ligands or antibodies that trigger CD16 (8, 12). Still, an in-depth understanding of the distinct functional roles of CD56bright and CD56dim NK cells and how those are enabled by differences in their receptor repertoires is lacking.

Human individuals bear approximately 6,000–30,000 different NK-cell phenotypes, and >100,000 different NK-cell phenotypes are present on a larger population level (13). The discovery of this high degree of cell-to-cell variability in a given NK-cell population has led to a growing appreciation for the remarkable phenotypic and functional heterogeneity of NK cells. Coinciding, multiple specialized, tissue-resident NK-cell subsets have been discovered in various tissues that fulfil distinct tissue-specific functions and are characterized by unique phenotypes (8, 14). Nevertheless, the molecular determinants of NK-cell-mediated recognition of aberrant cells remain incompletely understood. Given the close link between surface expression and immune functions in NK cells, further in-depth phenotypic characterization of NK cells is a prerequisite in gaining a better insight of how NK cells mediate their effector responses. These efforts may subsequently assist in identifying highly functional NK-cell subsets recognizing virus-infected or malignant cells. Thus, we aimed to comprehensively characterize the phenotypic diversity of human NK cells and the repertoire of surface molecules that NK cells utilize to interact with their environment. Using a flow cytometry-based approach, we assessed the surface expression of 338 molecules on human peripheral blood NK cells. This allowed detailed phenotypic characterization of primary NK cells, the assessment of inter-individual variability in receptor surface expression and comparative analyses between CD56bright and CD56dim NK cells.

## Methods

### Peripheral Blood Sample Acquisition and Processing

Citrate-treated peripheral blood samples were obtained from healthy blood donors recruited at the Institute for Transfusion Medicine, University Medical Center Hamburg-Eppendorf, Hamburg, Germany. Peripheral blood mononuclear cells (PBMCs) were isolated by density-gradient centrifugation, washed, and subsequently resuspended in complete medium (RPMI 1640 Medium, Thermo Fisher Scientific, Waltham, MA, USA) supplemented with 10% (v/v) fetal bovine serum (FBS, Biochrom GmbH, Berlin, Germany), 100 U/ml penicillin and 100 µg/ml streptomycin (Sigma Aldrich, St. Louis, MO, USA).

### Enrichment of NK Cells

Primary NK cells were enriched from PBMCs using an immunomagnetic negative selection strategy (EasySep Human NK cell Isolation Kit, Stemcell Technologies, VA, Canada) according to the manufacturer’s protocol. Enriched NK cells were suspended in complete medium at a density of 5 × 10^6^ cells/ml and cultured overnight in the presence of low-dose recombinant human IL-15 (5 ng/ml) and recombinant human IL-2 (50 U/ml) at 37 °C, 5% (v/v) CO_2_.

### Antibodies and Flow Cytometry

Multi-parameter flow cytometry was used for phenotypic and functional characterization of NK cells. Cells were acquired using a BD LSRFortessa flow cytometer (BD Biosciences, Franklin Lakes, NJ, USA). Data was further analyzed using FlowJo 10.6.1 software (FlowJo, Ashland, OR, USA). A comprehensive list of all antibodies and reagents is provided in **Supplementary Table 1**. The corresponding gating strategy is displayed in **Supplementary Figure 1**. Gating on educated and uneducated NK cells was based on HLA class I genotyping (**Supplementary Table 2**).

### Multiplexing and High-Throughput Phenotypic Analysis

Expression of up to 338 individual surface molecules was assessed using flow cytometry. Enriched primary peripheral blood NK cells were washed and resuspended in DPBS (Sigma Aldrich, St. Louis, MO, USA) supplemented with 1 mM EDTA and 2% (v/v) FBS. Cells from up to four different donors were multiplexed using distinct combinations of α-hCD45 conjugated to BV605 or AF700 (donor 1: no AB, donor 2: AF700, donor 3: BV605, donor 4: AF700 + BV605) to allow for re-identification of NK cells from the respective donors. Additional surface staining for viability, expression of CD3, CD14, CD16, CD19, and CD56 as well as for the NK-cell receptors KIR2DL1, KIR2DL2/L3, KIR3DL1, NKG2A, NKG2C, and CD57 was performed. NK cells from different donors were washed with DPBS and then combined. Subsequently, cells were stained with pre-titrated APC-conjugated antibodies for a total of 384 surface antigens and controls (MACS marker screen, human, Miltenyi Biotec, Bergisch Gladbach, Germany) following the manufacturer’s instructions. Surface expression was assessed as relative frequency of NK cells positive for each marker as well as median fluorescence intensity (MFI) of each marker within the respective subset. Placement of gates was based on FMO controls (**Supplementary Figure 1**).

### Statistical Analysis

Data of cell populations (% of positive cells), for which the parent population (denominator of the %) was <100 cells were set as missing, and analyses performed on available (non-missing) data. Data presentation of individual antigens and sub-populations in figures may therefore deviate from the reported overall number of individuals. Hierarchical clustering and principal component analyses were used to describe the relationships between cell populations. For the analyses of bulk NK cells, only cell populations with an inter-donor range of ≥5 percentage point (p.p.) were included in these analyses, the other cell populations being considered of little interest for the research question due to their low inter-donor variability in surface expression. Chi-square test was used for comparison of overall expression on CD56bright and CD56dim NK cells. Paired comparisons of CD56bright and CD56dim NK-cell subsets were done with the Wilcoxon signed rank test and adjusted for multiplicity using the Benjamini and Hochberg false discovery rate (FDR) (15). FDR-adjusted p-values <0.05 were considered statistically significant, and a median difference of >5% between subsets was considered of biological interest. Categorization of surface molecules into functional groups was based on manual annotation using GO identifiers (16, 17). Spearman’s ρ was used for correlation of median MFI and median % of positive NK cells. Wilcoxon signed rank test without multiplicity adjustment was used for paired comparison of the MFI of HLA-E between CD56bright and CD56dim NK cells.

Analyses were performed using R software (The R foundation, Vienna, Austria), version 3.6.1, and GraphPad Prism (GraphPad Software, La Jolla, CA, USA), version 8.2.1.

## Results

### Aim of the Study and Overview of Workflow

**Figure 1:**
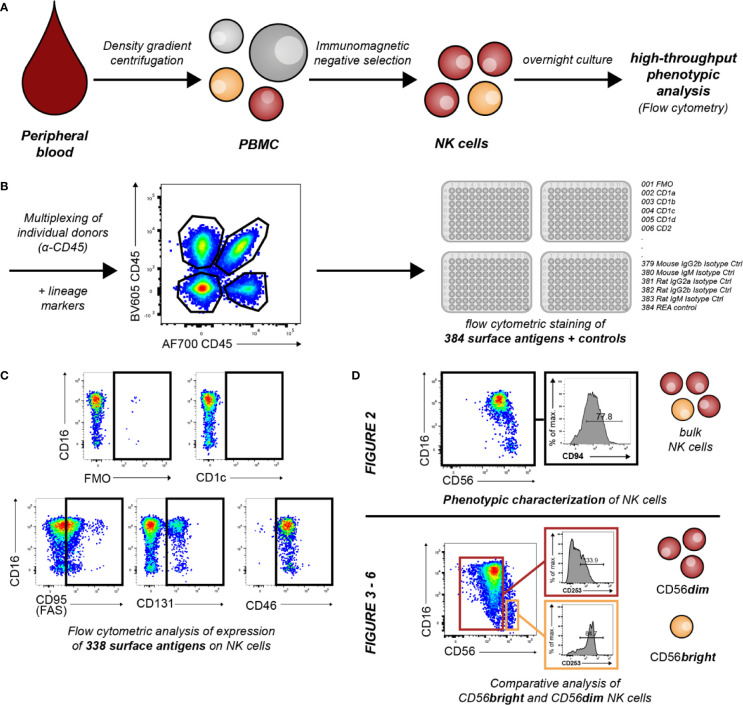
Overview of workflow and conducted experiments. **(A)** Primary human NK cells from peripheral blood of healthy donors were enriched by density gradient centrifugation and subsequent immunomagnetic negative selection. Enriched cells were cultured overnight in low-dose IL-2 (50 U/ml) and IL-15 (5 ng/ml). **(B)** For flow cytometric identification and purity assessment of NK cells, cells were stained for the expression of the lineage markers CD3, CD14, CD19, CD16, and CD56. For further discrimination of educated and uneducated sub-populations, NK cells were additionally labeled with antibodies against NKG2A, KIR3DL1, KIR2DL1, and KIR2DL2/L3. NK cells from up to four individual donors were subsequently multiplexed for further NK-cell phenotyping using α-hCD45. Multiplexed NK cells were stained with 384 antibodies and controls against surface antigens (n = 18). **(C)** Controls and surface antigens used for identification of NK cells and their subsets were excluded from further analysis. Therefore, expression of 338 surface antigens was assessed on NK cells. Exemplary dot plots show differential patterns of expression. **(D)** Downstream analyses focused on phenotypic characterization of NK cells and assessment of inter-individual variability in surface expression (**Figure 2**) as well as phenotypic differences between CD56bright and CD56dim NK cells (**Figures 3**–**6**).

**Figure 2:**
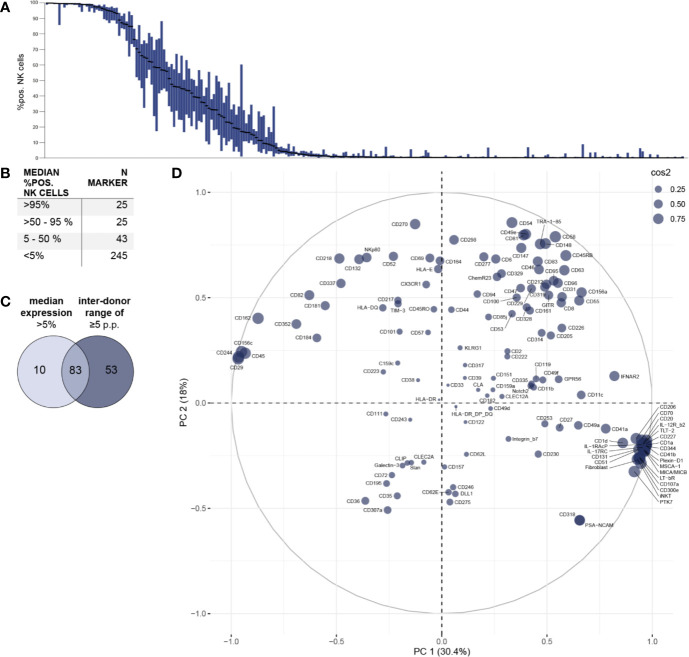
Phenotypic characterization of bulk NK cells and assessment of inter-individual variability. Surface expression of 338 different surface molecules on NK cells was assessed using flow cytometry (n = 18). **(A)** Distribution of expression of surface antigens on NK cells displayed as % positive NK cells. Bar graphs show IQR of expression of the respective surface molecule and black bars indicate the median. Surface molecules were ranked descending by median expression. **(B)** Summary table shows numeric results for overall surface expression. A total of 93 surface molecules exhibited a median expression of >5% of NK cells. **(C)** Venn diagram displaying the number of markers identified on NK cells with a median expression of >5% and/or a minimum inter-donor range of ≥5 p.p. **(D)** Principal component analysis was performed on all 136 surface antigens with a minimum inter-donor range of ≥5 p.p. (i.e. on all molecules with inter-individual variability in surface expression). Circle size indicates quality of representation (cos2) of each surface molecule.

**Figure 3:**
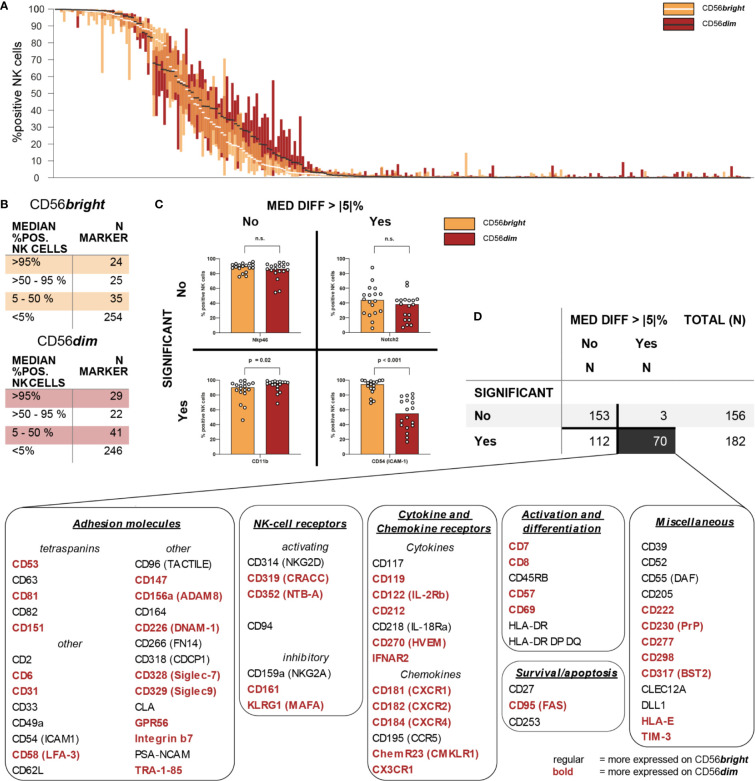
Intra-individual differences in surface expression between CD56bright and CD56dim NK-cell subsets. Expression of 338 different surface antigens on CD56bright and CD56dim NK cells was assessed using flow cytometry (n = 18). **(A)** Overall distribution of surface expression on CD56bright and CD56dim NK-cell subsets displayed as % positive NK cells. Bar graphs show IQR of expression of the respective molecule and white/black bars indicate the median. Surface antigens were ranked descending by median expression for each subset separately, thus their orders are not identical. **(B)** Summary tables show numeric results for overall surface expression on CD56bright and CD56dim NK cells. Eighty-four and 92 surface molecules were expressed on CD56bright and CD56dim NK cells, respectively (>5% median expression). A total of 104 surface antigens were consistently expressed on at least one of these NK-cell subsets. **(C)** Expression of all 338 surface antigens was compared between CD56bright and CD56dim NK cells. Wilcoxon signed rank tests were performed between subpopulations with an FDR adjustment for test multiplicity. Data was analyzed regarding median differences in expression >5 p.p. and statistical significance (FDR-adjusted p < 0.05). Representative bar graphs show exemplary data for all possible combinations of the two criteria. **(D)** Summary table shows numeric results. All 70 surface molecules with statistically significant differences >5 p.p. in expression between CD56bright and CD56dim NK cells were grouped according to their function.

**Figure 4:**
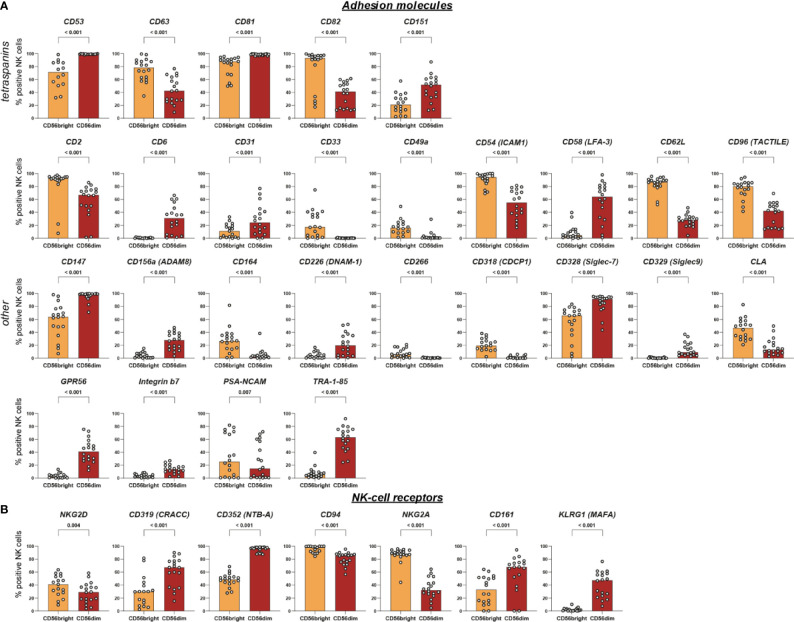
Intra-individual differences in surface expression of adhesion molecules and NK-cell receptors between CD56bright and CD56dim NK-cell subsets. NK cells were assessed for surface expression of 338 different surface antigens using flow cytometry (n = 18). Wilcoxon signed rank tests were performed between subpopulations with a Benjamini and Hochberg FDR adjustment for test multiplicity. Seventy molecules displayed a median difference >5 percentage points between CD56bright and CD56dim NK cells and were statistically significant (FDR-adjusted p < 0.05). Those 70 surface molecules were subsequently grouped according to their function. Bars indicate median expression (% positive NK cells) of all members of the groups titled adhesion molecules **(A)** and NK-cell receptors **(B)**.

**Figure 5:**
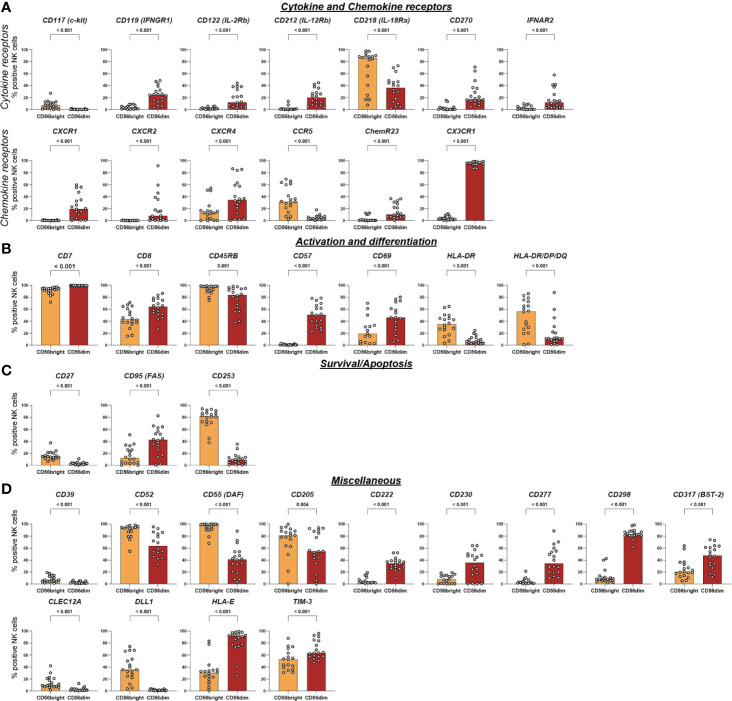
Intra-individual differences in surface expression of cytokine and chemokine receptors, activation and differentiation, survival/apoptosis, and miscellaneous molecules between CD56bright and CD56dim NK-cell subsets. NK cells were assessed for surface expression of 338 different surface antigens using flow cytometry (n = 18). Wilcoxon signed rank tests were performed between subpopulations with a Benjamini and Hochberg FDR adjustment for test multiplicity. Seventy molecules displayed a median difference >5 percentage points between CD56bright and CD56dim NK cells and were statistically significant (FDR-adjusted p < 0.05). Those 70 surface molecules were subsequently grouped according to their function. Bars indicate median expression (% positive NK cells) of all members of the groups titled cytokine and chemokine receptors **(A)**, activation and differentiation **(B)**, survival/apoptosis **(C)**, and miscellaneous **(D)**.

**Figure 6:**
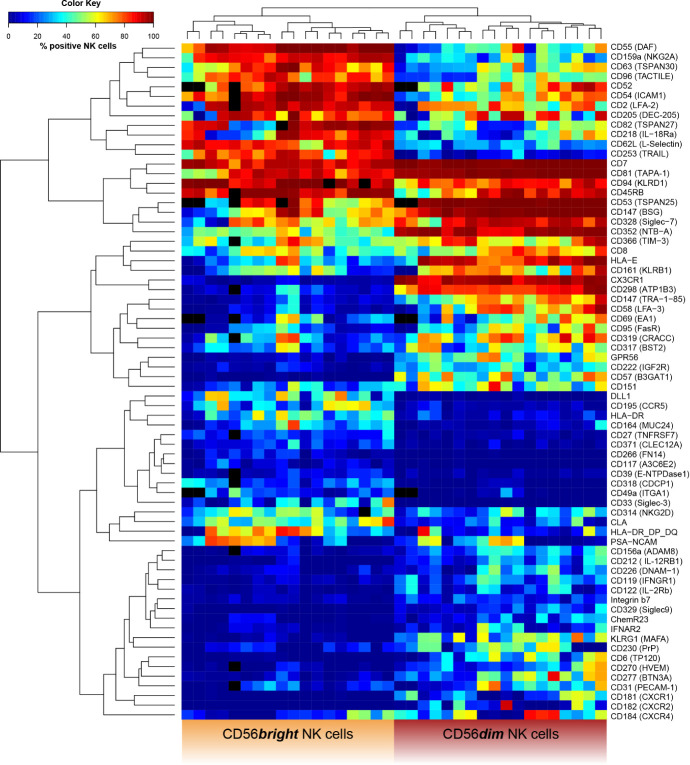
Receptor expression-based unsupervised hierarchical clustering of CD56bright and CD56dim NK cells. NK cells were assessed for surface expression of 338 different surface antigens using flow cytometry (n = 18). Wilcoxon signed rank tests were performed between subpopulations with a Benjamini and Hochberg FDR adjustment for test multiplicity. Seventy molecules displayed a median difference >5 p.p. between CD56bright and CD56dim NK cells and were statistically significant (FDR-adjusted p < 0.05). Heatmap displaying frequency of surface antigen-expressing NK cells based on these 70 molecules in CD56bright (left) and CD56dim NK cells (right) for each donor. Field color indicates median surface expression (in %) on the respective NK-cell subset. Black fields represent missing or excluded data (i.e. due to insufficient cell population size <100 cells). Unsupervised hierarchical clustering confirmed the major phenotypic differences as data clearly clustered into CD56bright and CD56dim NK cells based on expression of surface molecules.

The aim of the study was to comprehensively characterize the phenotypic diversity of human NK cells in order to gain new insights into the receptor repertoires NK cells utilize to interact with their environment and to mediate their effector functions. As depicted in **Figure 1**, enriched human primary peripheral blood NK cells from multiple donors were multiplexed using α-hCD45 and then stained for 384 individual surface antigens and controls in a high-throughput manner using flow cytometry. Controls and surface antigens used for identification of NK cells and their subsets were excluded from further analysis (**Supplementary Table 1**). Therefore, this approach allowed comparison of the surface expression of 338 molecules on NK cells. We observed multiple different receptor-specific expression patterns among NK cells (**Figure 1C**). This included the complete lack of receptor expression (e.g. CD1c), binary expression patterns (e.g. CD131) as well as expression with differential surface densities on all NK cells (low: e.g. CD95, high e.g. CD46). Downstream analyses focused on phenotypic characterization of bulk NK cells and inter-individual variability in expression (**Figure 2**) as well as comparative analysis between the two major CD56bright and CD56dim NK-cell subsets (**Figures 3**–**6**). Median fluorescence intensity (MFI) and relative frequency (% positive NK cells) for each surface antigen were determined as parameters of expression. Spearman’s ρ confirmed a significant correlation between both parameters (**Supplementary Figure 2**, *r*_s_ = 0.86, p < 0.0001). Main statistical analyses were conducted using relative frequency (% positive NK cells), therefore the main manuscript displays figures showing relative frequency of expression data. Similar figures showing MFI are presented in the data supplement as indicated.

### NK Cells Express at Least 146 Different Surface Molecules

First, the overall expression of the 338 tested surface antigens on bulk NK cells was assessed (**Figure 2A**). Data for all 338 surface markers (including MFI) on bulk NK cells as well as CD56bright and CD56dim NK-cell subsets is compiled in **Supplementary Table 3**. Using a threshold of 5% median percentage of positive NK cells for each surface antigen, we identified 93 molecules expressed on NK cells. Out of those, 25 antigens had a median percentage of >95% positive NK cells. Another 25 molecules were expressed in median on the majority of NK cells and 43 antigens to a lesser degree (**Figure 2B**).

In addition to identifying surface molecules with expression on NK cells using a strict cutoff, we selected all 136 surface molecules with a minimum inter-donor range of ≥5 p.p. for subsequent principal component analysis. The majority of these markers (83 out of 136) were expressed above the 5% median percentage cut off (**Figure 2C**). However, 53 markers with a minimum inter-donor range of ≥5 p.p. had a median expression of less than 5%. This selection criterion of minimum inter-individual variability in expression allowed identification of surface molecules that may contribute to differences in NK-cell phenotypes and functionality between individuals. The relationship between these 136 surface molecules and their respective impact on overall phenotypic variability are shown in **Figure 2D**. While markers with higher median expression tend to be located in the upper part of the PCA plot, surface antigens with low median expression are preferentially plotted in the lower part of the PCA. In the particular, some of the latter form a clear cluster with increased impact on the overall phenotypic variability. Despite excluding surface antigens from further analysis that were uniformly expressed on all NK cells using this threshold of minimum inter-donor range, we identified an increased number of molecules compared to using the cutoff of 5% median expression. Among those 53 additional receptors were TLT-2, LAG-3 (CD223), CD70, CD27 (TNFR), and CD39 which have been implicated in various NK-cell functions (e.g. target cell recognition).

Finally, we investigated receptors exhibiting very high variability in surface expression between individuals. Forty-seven surface molecules demonstrated an inter-donor range of >50 p.p. and the largest ranges of expression were observed for surface molecules CD58 (LFA-3) (range 95 p.p.), CD205 (range 94 p.p.), CD161 (range 93 p.p.), CXCR2 (range 86 p.p.), and CD2 (range 85 p.p.).

Combining our two selection criteria (median expression >5% or inter-donor range ≥5 p.p.), we detected a total of 146 molecules on NK cells (**Supplementary Figure 3**). Only 10 of those did not meet our inter-donor range criterion which emphasizes how common inter-individual variability in NK-cell receptor expression is.

### CD56bright and CD56dim NK Cells Express Similar Numbers of Receptors

Next, we assessed the overall expression of surface antigens on the two major NK-cell subsets CD56bright and CD56dim NK cells.

Using a threshold of 5% median percentage of positive NK cells for each of the surface antigens, we observed expression of 84 and 92 molecules on CD56bright and CD56dim NK cells, respectively. CD56bright and CD56dim NK cells displayed similar patterns of overall distribution (**Figure 3A**). Twenty-four and 29 antigens were in median expressed on >95% of CD56bright or CD56dim NK cells, respectively. Another 25 and 22 molecules were expressed on the majority of NK cells from both subsets. Additionally, 35 and 41 antigens were expressed on CD56bright and CD56dim NK cells to a lesser degree (**Figure 3B**). Overall, these differences were not statistically significant (χ^2^ = 1.265, p = 0.73). These data indicate that both CD56bright and CD56dim NK cells express very similar numbers of surface receptors. A total of 104 surface molecules was expressed on at least one of the major NK-cell subsets.

### CD56bright and CD56dim NK Cells Are Differentially Equipped With Surface Receptors

Subsequently, we aimed to characterize the phenotypic differences between the two major NK-cell subsets by comparing the expression of all 338 surface antigens on CD56bright and CD56dim NK cells. We identified 70 molecules with statistically significant differences in expression between the two NK-cell subsets which were defined as median difference in surface expression >5 p.p. with an FDR-adjusted p < 0.05 (**Figure 3C**). Median differences within the positive range indicate higher expression on CD56dim NK cells whereas negative values represent a higher expression on CD56bright NK cells. Only 34 out of the 104 receptors expressed on at least one of the subsets were not differentially expressed between CD56bright and CD56dim NK cells. Those included activating NCR NKp46, co-stimulatory 2B4 and adhesion molecules CD18 and CD11a (the two subunits of LFA-1). The majority of receptors—70 out of 104—exhibited differential surface expression between the two subsets.

All 70 markers with differential expression were categorized into six groups according to their function—adhesion molecules, NK-cell receptors, cytokine and chemokine receptors, survival/apoptosis, activation and differentiation, as well as miscellaneous (**Figure 3D**). This highlights how phenotypic differences between the two major NK-cell subsets associate with a broad range of NK-cell functions [**Figures 4**, **5**, **Supplementary Figure 4** (MFI)].

Several of the 70 surface molecules with differential expression are known to be predominantly expressed by either CD56bright [e.g. CD253 (TRAIL) (median difference −72 p.p., p < 0.0001), CD55 (DAF) (−58 p.p., p < 0.0001), CD62L (−55 p.p., p < 0.0001), NKG2A (−53 p.p., p < 0.0001)] or CD56dim NK cells [e.g. CX3CR1 (91 p.p., p < 0.0001), CD57 (51 p.p., p < 0.0001), CXCR1 (20 p.p., p < 0.0001)]. Moreover, we identified multiple surface molecules that were not previously associated with predominant expression on either CD56bright [e.g. CD82 (median difference −41 p.p., p < 0.0001)] or CD56dim NK cells [e.g. CD298 (72 p.p., p < 0.0001), CD58 (LFA-3) (56 p.p., p < 0.0001), CD147 (35 p.p., p < 0.0001), HLA-E (55 p.p., p < 0.0001)].

Given the observed differences in receptor expression, we tested whether CD56bright and CD56dim NK cells can be clearly classified based on expression of these surface molecules. Unsupervised hierarchical clustering confirmed the major phenotypic differences between the cell subsets [**Figure 6** and **Supplementary Figure 5** (MFI)]. Our data clustered into CD56bright and CD56dim NK cells. A similar analysis comparing uneducated and educated NK cells did not show clustering based on expression of surface molecules (**Supplementary Figure 6**).

Overall, our high-throughput phenotypic analysis revealed the major phenotypic differences between CD56bright and CD56dim NK cells among receptors with a broad range of functions that were confirmed by unsupervised hierarchical clustering.

## Discussion

Our comprehensive phenotypic characterization of NK cells revealed relevant expression of 146 surface molecules on bulk NK cells and considerable variability in expression for 136 molecules between individual donors. Of those, 53 showed a donor-specific expression and were only detected on NK cells in a subset of individuals. Among these receptors were CD27 (TNFR) and its ligand CD70, CD39, LAG-3, and TLT-2. Expression of the receptor CD27 on subpopulations of NK cells and its influence on target cell recognition were already reported in the 1990s (18). More recently, reverse signaling *via* its ligand CD70 was shown to also enhance NK-cell effector functions and contribute to tumor immunosurveillance (19). CD39 is an ectonucleotidase that contributes to high levels of adenosine in the tumor microenvironment where adenosine functions as an immunosuppressive metabolite and regulates tumor immunosurveillance. Expression of CD39 was reported for tumor infiltrating lymphocytes and, to a lesser degree, resting NK cells. Currently, multiple substances targeting CD39—i.e. monoclonal antibodies and enzyme inhibitors—are investigated as potential therapeutic options for metastasized solid tumors as CD39 enzyme activity seems especially relevant for NK-cell-mediated control of metastases (20, 21). Those examples clearly show how inter-individual differences in NK-cell phenotypes may underlie differential abilities to control certain diseases. However, the function and biological significance of some molecules with inter-individual differences in expression, such as LAG-3 and TLT-2, remain rather elusive. LAG-3 is an established surface marker for NK-cell exhaustion and currently under investigation as a target for checkpoint inhibition in tumor immunotherapy. As most data on NK-cell exhaustion was generated in the context of anti-tumor immunity, little is known about LAG-3+ NK cells in healthy donors (22, 23). We observed expression of LAG-3 on NK cells in only three out of 18 donors (5.23, 6.95, and 11.7% expression, respectively). One possible explanation for the presence of exhausted NK-cell subsets within otherwise healthy individuals may be an ongoing, asymptomatic viral infection or virus reactivation. Likewise, the function of TLT-2 on NK cells has not been investigated yet. TLT-2 is a TREM family receptor expressed on granulocytes, monocytes, and B cells. The receptor is involved in multiple immunological processes such as neutrophil chemotaxis, regulation of terminal neutrophil effector functions, and phagocytosis of apoptotic cells by macrophages (24).

The highest inter-individual variability in expression was observed for the surface molecules CD2, CD58 (LFA-3), CD161, and CD205. Several of these are known to participate in recognition of aberrant cells or mediate other NK-cell functions. For example, CD58 and CD2 are a pair of reciprocal adhesion molecules mediating heterotypic and homotypic intercellular recognition. CD2 is well established as one of the major costimulatory NK-cell receptors binding to CD58 expressed on target cells (25). Additionally, CD2 and CD58 also contribute to NK-cell cross-talk by providing costimulatory signals among NK cells themselves which regulate the development of NK-cell effector functions (26). However, little is known about the functional impact of differential expression of CD58 and CD2 between individuals. Notably, we observed no correlation for expression of CD2 and CD58 within individual donors. CD161 is an inhibitory NK-cell receptor that binds to its ligand LLT1 on—predominantly activated—B cells and DCs (27). Expression of CD161 decreases with aging as well as following HCMV infection which may explain some of the inter-individual variability we observed (28). As all blood samples used in our study were donated anonymously, i.e. without information on age, gender, or CMV-serostatus, we were unable to investigate the influence of these factors on the observed differences in surface expression. CD205 is an antigen uptake receptor commonly used for phenotypic identification of DCs and is associated with induction of immune tolerance in DCs (29). NK cells express CD205 on their surface but its functional impact for NK cells remains uncertain (30).

Altogether, the observed inter-individual differences of these particular receptors may lead to differential NK-cell functionality and thus potentially contribute to a diverging ability to control viral infections or malignancies.

To address the differences in the receptor repertoire of NK cells within each individual and between major NK-cell subsets, we characterized the phenotypes of CD56bright and CD56dim NK cells. Seventy molecules were differentially expressed by these subsets and associated with a broad range of NK-cell functions. Moreover, unsupervised hierarchical clustering confirmed major phenotypic differences between CD56bright and CD56dim NK cells. This resembles recent transcriptome data on peripheral blood NK cells that demonstrated clustering of CD56bright and CD56dim NK cells as well (31, 32). Interestingly, unsupervised hierarchical clustering did not result in the discrimination of NK cells into functionally divergent educated and uneducated NK-cell subsets. These observations are in line with recent investigations into the transcriptional profiles of educated and uneducated NK cells (33). A possible explanation for the lack of clear phenotypic and transcriptional differences between educated and uneducated NK cells is that NK-cell education does not alter receptor repertoires to a currently detectable degree and that changes induced by education primarily affect intracellular signaling (33).

Among the known functional differences between CD56bright and CD56dim NK cells is their response to different types of input stimuli: CD56dim NK cells are more prone to respond to cell surface ligands encountered in cell-cell interactions while CD56bright NK cells rather respond to soluble ligands such as cytokines and chemokines produced by other immune cells (4). Our results confirm the predominant expression of several surface molecules involved in mediating cell-cell interactions on CD56dim NK cells (e.g. CD31, ADAM8, CD226, GPR56, CD53, CD81, and CD151).

Furthermore, we discovered differential expression between CD56bright and CD56dim NK cells for CD58, CD147, HLA-E, and CD82 which were, to our best of knowledge, not reported before. CD58 (LFA-3) and CD147 both mediate intercellular recognition and were predominantly expressed on CD56dim NK cells that primarily recognize ligands bound to cell-surfaces (26, 34). Notably, CD58 expression was not only different between the two major NK-cell subsets but also associated with great variability between different individuals as discussed above. HLA-E is a non-classical HLA class I molecule that mediates effector responses of NK and T cells by presentation of leader peptides derived from classical HLA class I as well as pathogen- or stress-associated peptides. Engagement of HLA-E with activating receptor CD94/NKG2C may trigger NK-cell effector functions while engagement with CD94/NKG2A delivers inhibitory signals to NK cells (35). Additionally, signaling *via* CD94/NKG2A is one of the two major routes to facilitate NK-cell education i.e. to shape functional capacities of NK cells *via* certain self-reactive inhibitory receptors (36). Inter-individual differences in expression depending on certain genetic polymorphisms as well as altered expression in several disease settings—e.g. viral infections or malignant tumors—are well established (35, 37, 38). In contrast, to our best knowledge no data on differential expression by NK-cell subsets has been published. CD82 is a tetraspanin associated with inhibitory signaling and limiting cytotoxicity in NK cells. Generally, tetraspanins organize surface proteins into a highly organized network that is composed of different molecules and participates in various functions in each cell type (39, 40). Little is known though about the components and functions of the network in NK cells—and even less on differences between CD56bright and CD56dim NK cells. CD82 was primarily expressed on CD56bright NK cells. Another tetraspanin, CD81, is involved in NK-cell recruitment towards chemokines and cytokines (41). If CD82 fulfilled a similar role as CD81 in NK cells, predominant expression of CD82 might contribute to the ability of CD56bright NK cells to sensitively respond to cytokines and chemokines. Analogous functional properties for CD81 and CD82 in NK cells were demonstrated before as well as involvement of CD82 in chemokine-induced migration of DCs (39, 42).

Only 34 of the 104 surface molecules expressed on at least one of the major NK-cell subsets were not differentially expressed between CD56bright and CD56dim NK cells. Those receptors with equal expression on both subsets included activating NCR Nkp46, costimulatory 2B4 and adhesion molecules CD18 and CD11a (the two subunits of LFA-1) which were expressed on virtually all NK cells. Equipment of NK cells with these receptors may ensure a minimum level of activation potential regardless of NK-cell subset and thus be a prerequisite for all NK cells to fulfill their designated immune functions.

Altogether, we confirmed established differences in the receptor repertoire of CD56bright and CD56dim NK cells and identified additional surface molecules that contribute to the vast phenotypic differences of the major NK-cell subsets. Intriguingly, all of those were already implied in various immune functions for other cell types that allowed us to hypothesize upon possible functional implications for CD56bright and CD56dim NK cells.

Our flow cytometry-based phenotypic analysis provided new insights into the vast diversity of NK-cell phenotypes and shed light onto inter-individual differences in receptor profiles utilized by NK cells. Limitations of our approach include a rather high amount of NK cells required for the analysis of NK-cell sub-populations and potential alterations of certain receptors during sample processing (i.e. density centrifugation, overnight culture, use of cytokines). Nevertheless, our findings contribute to a better understanding of the phenotypic differences underlying differential functionality of NK cells on an inter-individual level, which determine e.g. different disease susceptibilities or differential abilities to control malignancies. Additionally, we further characterized the two major NK-cell subsets to better comprehend how the different functional roles of CD56bright and CD56dim NK cells are enabled by the variegated equipment with surface receptors. As our approach for phenotypic characterization is based on flow cytometry it may be used in conjunction with additional techniques to investigate other relevant NK-cell functions and thus be adapted to various fields of interest. Especially given the current developments in tumor immunotherapy, further insights into NK-cell-mediated recognition of aberrant cells may help in harnessing NK-cell properties for therapeutic strategies and clinical applications.

## Data Availability Statement

Raw data storage is performed by the Heinrich Pette Institute on an internal server. Raw data will be made available upon request and can be shared after confirming that data will be used within the scope of the originally provided informed consent. Phenotypic analysis data is compiled in a data supplement available with the online version of this article as indicated. R code used for statistical analyses will be also made available upon request.

## Ethics Statement

For this study, residual amounts of anonymized peripheral blood samples were used which were routinely taken from healthy blood donors and would have been discarded otherwise. All blood donors gave their general written consent to usage of their blood samples for scientific studies in an anonymized form. The anonymized use of human material complies with a vote by the ethics committee of the German Medical Association.

## Author Contributions

Conceptualization, methodology, CK. Investigation, VS. Statistical analyses, LR, VH-T, and VS. Statistical analyses—manuscript revision, VY and SV. Writing—original draft, VS and CK. Funding acquisition, CK and MA. Resources, MA, JS, AS, SP. All authors contributed to the article and approved the submitted version.

## Funding

SV was supported by the German Research Foundation (KO 5139/3-1).

## Conflict of Interest

The authors declare that the research was conducted in the absence of any commercial or financial relationships that could be construed as a potential conflict of interest.
