# Supplementary material for: Distinct Signatures in the Receptor Repertoire Discriminate CD56bright and CD56dim Natural Killer Cells
Source: Front Immunol. 2020 Dec 1;11:568927. doi: 10.3389/fimmu.2020.568927 (PMC7736243; doi:10.3389/fimmu.2020.568927)
Supplement: Supplementary file 1 [file DataSheet_1.pdf]

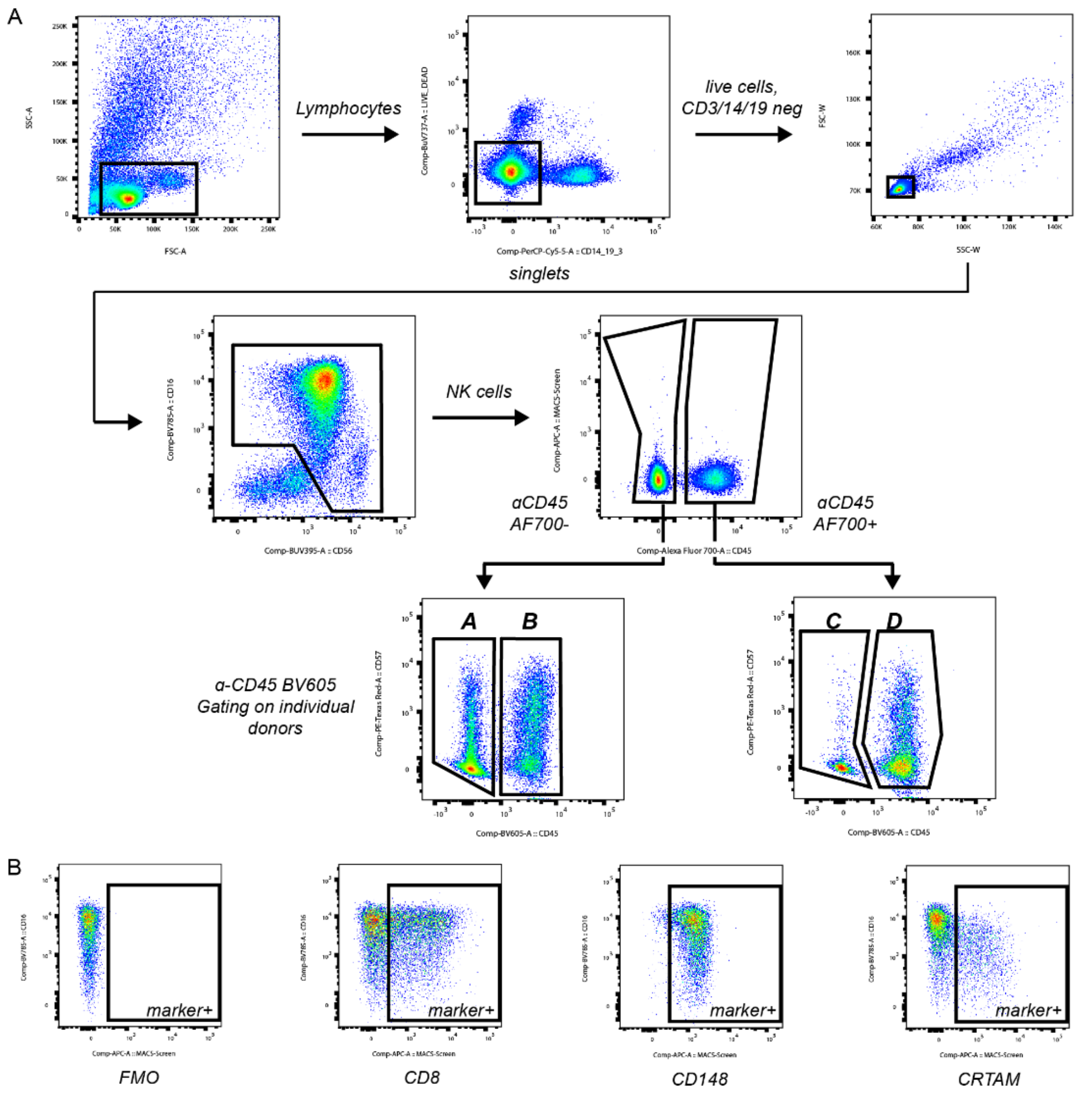

Supplementary Figure 1 | Flow cytometry gating strategy. **(A)** Gating of individual donor NK cells. Following the exclusion of dead cells, T cells, B cells, monocytes (LIVE/DEAD Blue, CD3, CD14, and CD19) and doublets, remaining single-cell lymphocytes were plotted against CD16 and CD56 to define NK cells. Identification of individual donor NK cells after multiplexing was conducted in a two-step way; first discriminating donor NK cells by the expression of CD45 using α-CD45-AF700 and subsequently by using α-CD45-BV605. **(B)** Gating for high-throughput phenotype analysis of 338 different surface molecules using an FMO control as a reference for NK cells lacking the respective surface antigen. Examples of expression patterns are shown.

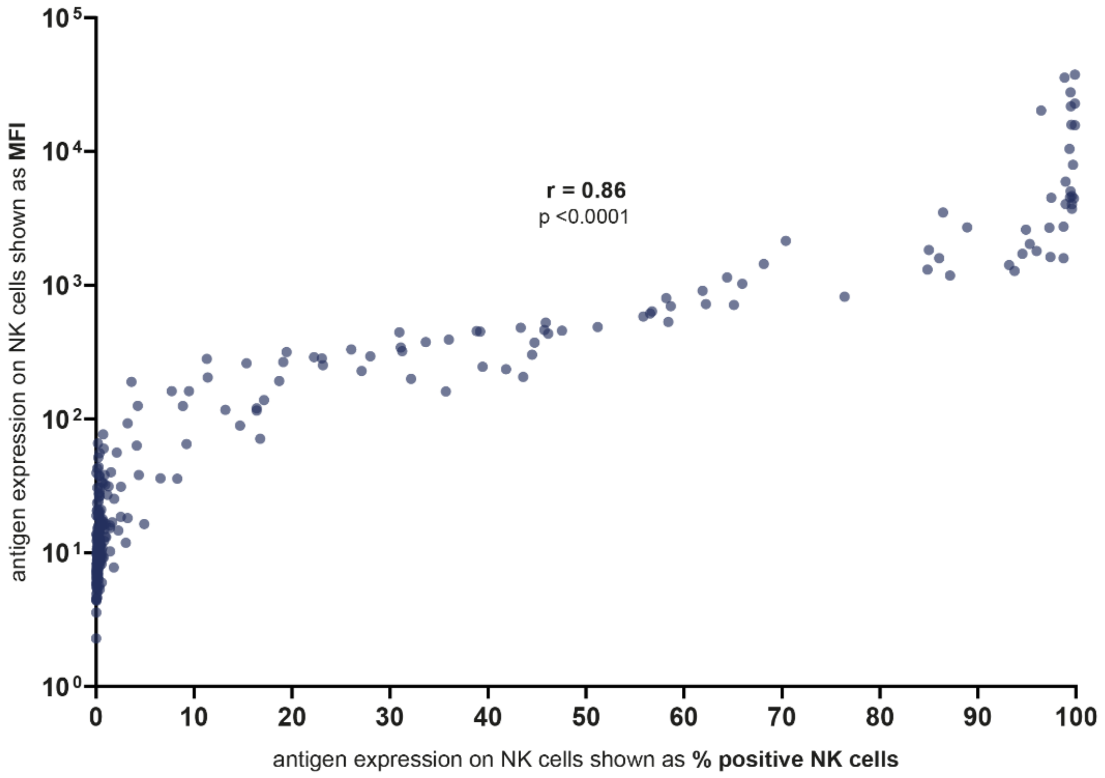

Supplementary Figure 2 | Correlation between median MFI and median relative frequency of antigen expression on primary NK cells. Primary NK cells were assessed for surface expression of 338 different surface antigens using flow cytometry. Expression was quantified as median fluorescence intensity (MFI) and as relative frequency of antigen-positive NK cells (in %); the median from all donors was calculated (n = 18). Spearman's ρ showed a positive correlation between median MFI (y-axis) and median % of positive NK cells (x-axis) and (r_s = 0.86, p < 0.0001).

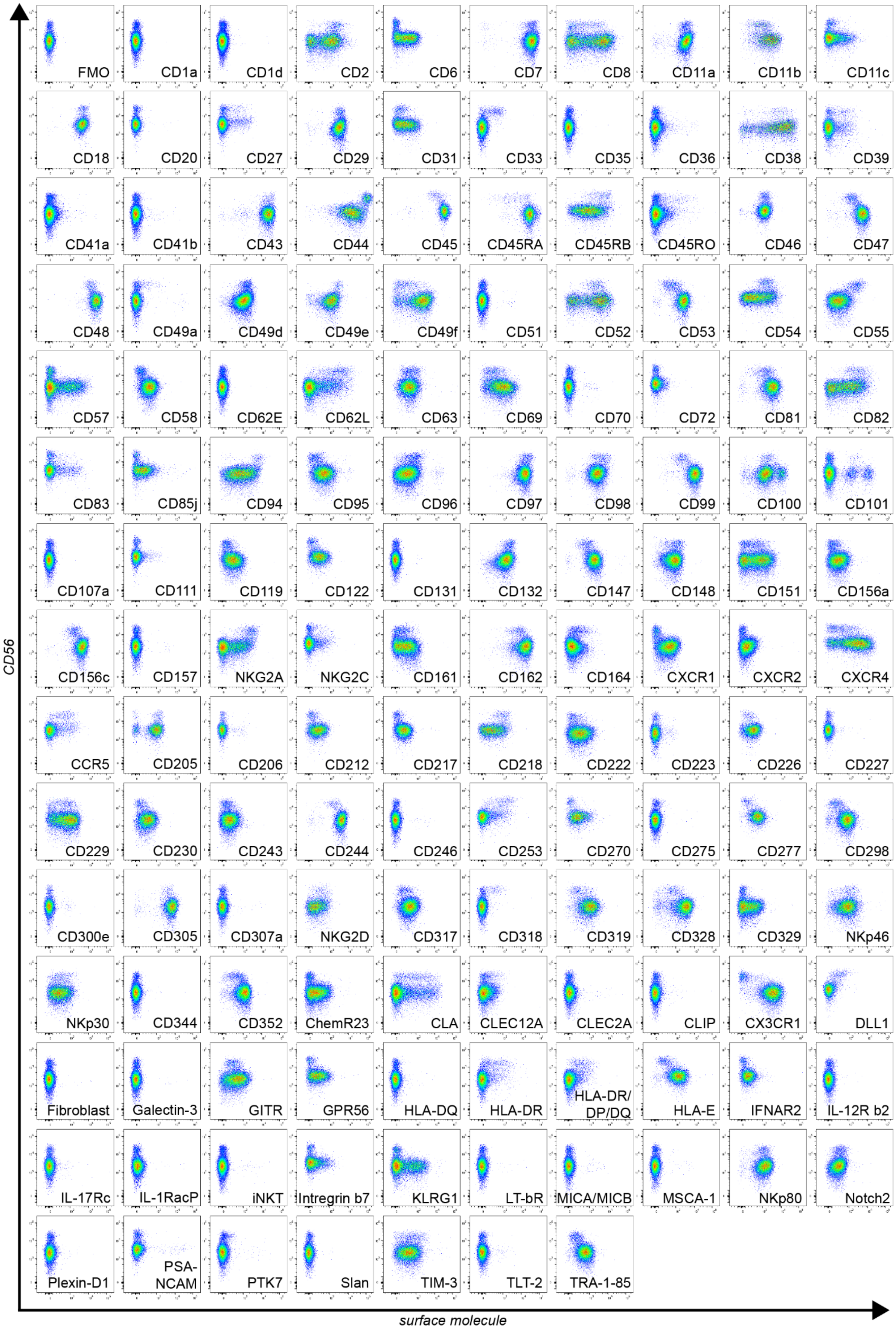

Supplementary Figure 3 | Expression patterns of surface molecules. Representative dot plots of all 146 surface molecules expressed on bulk NK cells. Expression was determined by using a combination of two selection criteria [median expression >5% or inter-donor range ≥5 percentage points (p.p.)].

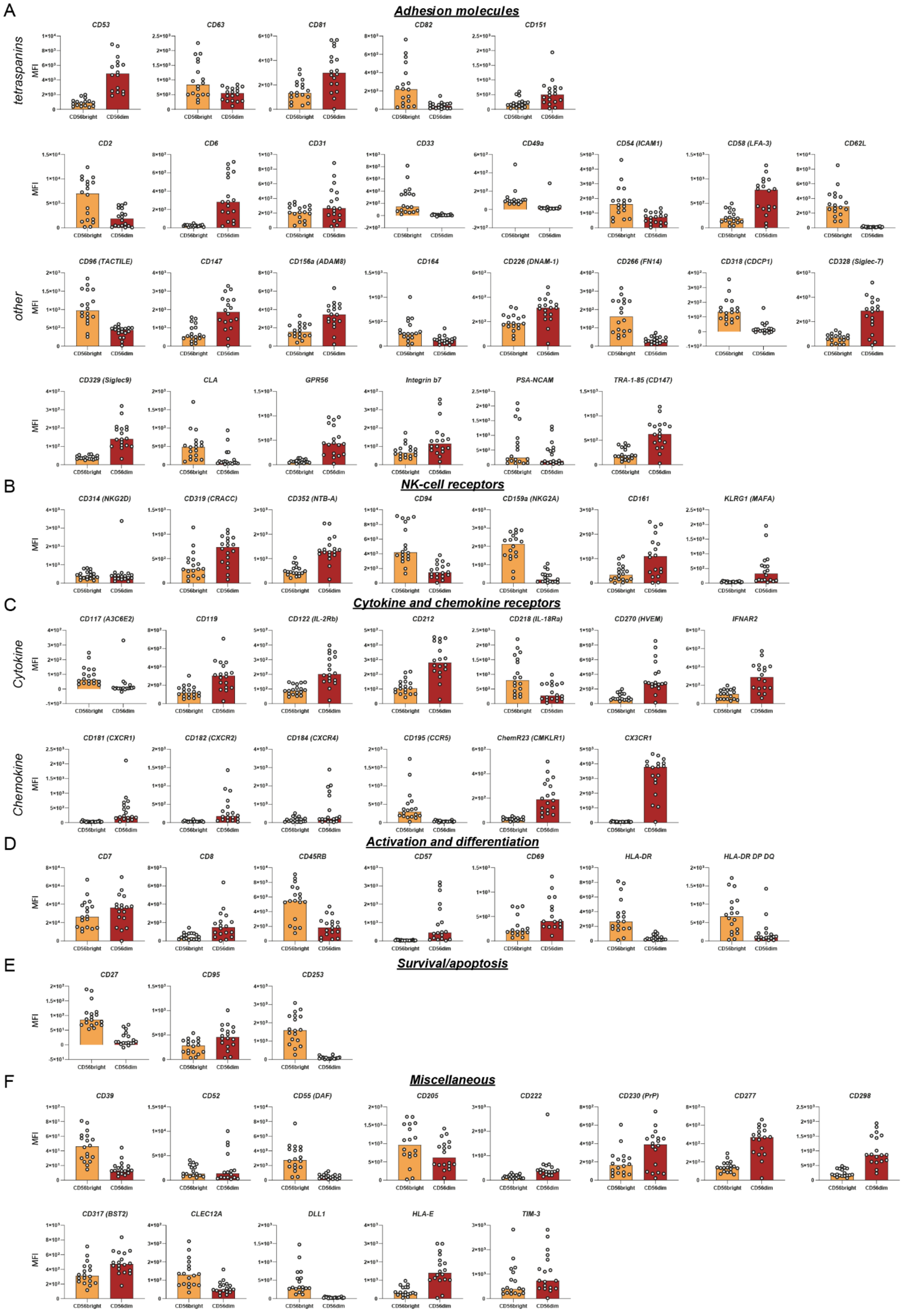

Supplementary Figure 4 | Intra-individual differences in surface expression (MFI) between CD56bright and CD56dim NK-cell subsets. Expression of 338 different surface antigens on CD56bright and CD56dim NK cells was compared using flow cytometry (n = 18). Statistical analysis (Wilcoxon signed rank tests with FDR-adjustment for multiplicity) based on % positive NK cells identified 70 molecules with differential expression between CD56bright and CD56dim NK cells. **(A-F)** All 70 surface molecules with statisticially significant differences (>5 p.p. and FDR-adjusted $p < 0.05$) were grouped according to their function. Bar graphs indicate the median MFI of the respective surface antigens.

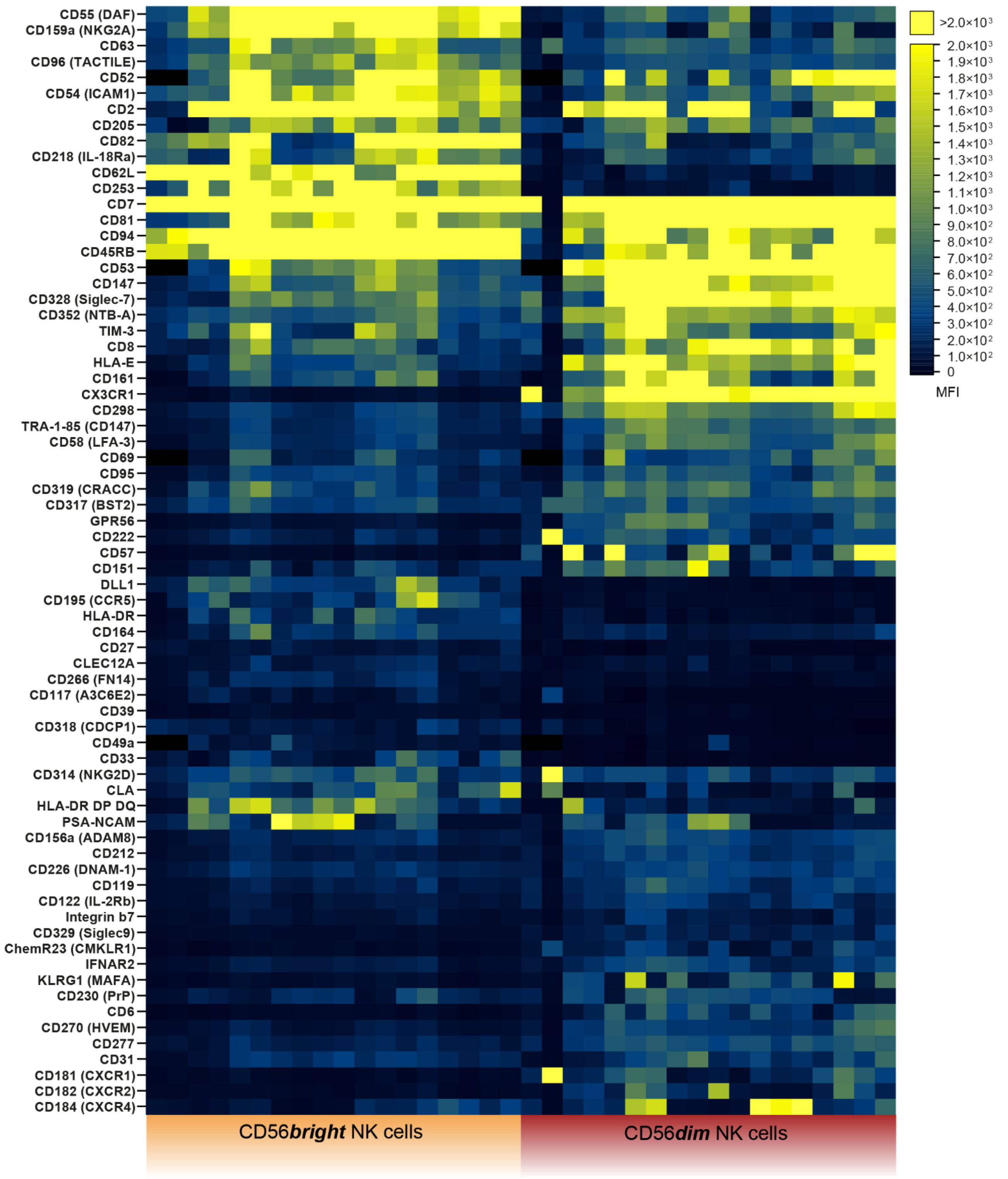

Supplementary Figure 5 | Differential expression of surface molecules on CD56bright and CD56dim NK cells (MFI). Expression of 338 different surface antigens on CD56bright and CD56dim NK cells was compared using flow cytometry (n = 18). Statistical analysis (Wilcoxon signed rank tests with FDR-adjustment for multiplicity) based on % positive NK cells identified 70 molecules with differential expression between CD56bright and CD56dim NK cells. All 70 surface molecules with statisticially significant differences (>5 p.p. and FDR-adjusted $p < 0.05$) were included for display in this heatmap. Field color indicates MFI on the respective NK-cell subset. Black fields represent missing data.

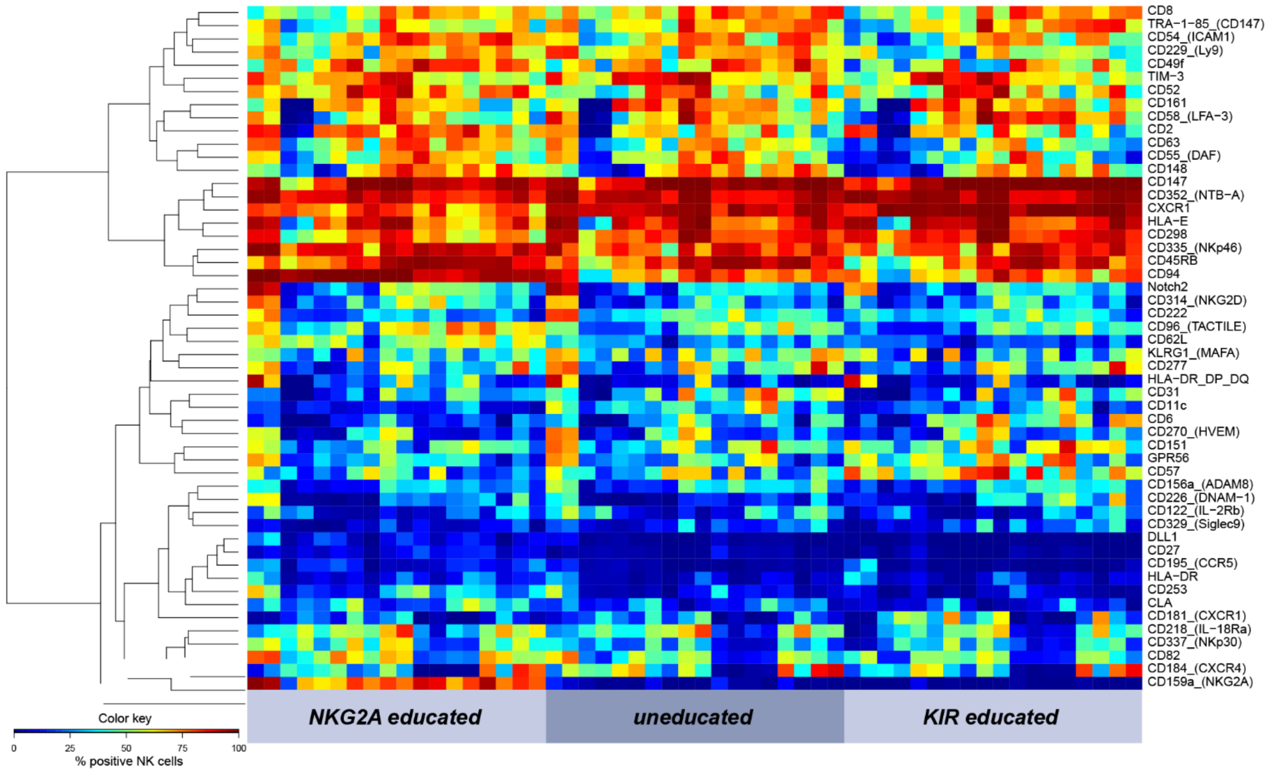

Supplementary Figure 6 | Receptor expression-based unsupervised hierarchical clustering of educated and uneducated NK cells. NK cells were assessed for surface expression of 338 different surface antigens using flow cytometry (n = 18). Wilcoxon signed rank tests were performed between subpopulations with a Benjamini and Hochberg FDR adjustment for test multiplicity. Heatmap displaying frequency of surface antigen-expressing NK cells based on all molecules with a median difference >5 percentage points and FDR-adjusted p < 0.05 between at least two of the NK-cell subsets. Field color indicates median surface expression (in %) on the respective NK-cell subset.

Supplementary Table 1. Key resources

Reagent or resource	*Conjugate*	*Clone*	*Source*	*Catalogue number*	*RRID*
Antibodies					
α-human CD56	BUV395	NCAM16.2	BD Biosciences	Cat#563554	AB_2687886
α-human CD16	BV785	3G8	BioLegend	Cat#302046	AB_2563803
α-human KIR2DL2/L3/S2	BV711	DX27	BD Biosciences	Cat#745442	AB_2742987
α-human CD45	BV605	2D1	BioLegend	Cat#368524	AB_2715826
α-human KIR3DL1	BV421	DX9	BioLegend	Cat#312714	AB_2561652
α-human CD14	PerCP-Cy5.5	HCD14	BioLegend	Cat#325621	AB_893252
α-human CD19	PerCP-Cy5.5	HIB19	BioLegend	Cat#302229	AB_2275547
α-human CD3	PerCP-Cy5.5	UCHT1	BioLegend	Cat#300430	AB_893299
α-human KIR2DL1/S5	FITC	143211	R&D Systems	Cat#FAB1844F-100	AB_2130402
α-human NKG2A	PE-Cy7	Z199	Beckman Coulter	Cat#B10246	AB_2687887
α-human CD57	PE-Dazzle	HNK-1	BioLegend	Cat#359620	AB_2564063
α-human KIR2DL2/L3/S2	PE	DX27	BioLegend	Cat#312606	AB_2130554
α-human NKG2C	PE	REA205	Miltenyi	Cat#130-103-635	AB_2655394
α-human CD45	AF700	2D1	BioLegend	Cat#368514	AB_2566374
Biological Samples					
Citrate-treated peripheral blood			University Medical Center Hamburg-Eppendorf, Hamburg, Germany		
Chemicals					
LIVE/DEAD Fixable Blue Dead Cell Stain Kit			Invitrogen	Cat#L23105	
RPMI 1640 Medium, no glucose			ThermoFisher	11879020	
Critical Commercial Assays					
MACS Marker Screen, human	APC		Miltenyi	Cat#130-110-055	
EasySep Human NK Cell Enrichment Kit			Stemcell	Cat#19055	
Software and Algorithms					
FlowJo software v.10.6.1			FlowJo, Ashland, OR, USA		
GraphPad Prism v.8.2.1			GraphPad Software Inc., La Jolla, CA, USA		
R Software v.3.6.1			The R foundation, Vienna, Austria		

Supplementary Table 2. HLA class I and KIR genotypes of all donors

Donor ID	*HLA-A*		*HLA-B*		Bw4/	*HLA-C*		HLA-C	*KIR2D#*											*KIR3D#*					KIR
	Allele 1	Allele 2	Allele 1	Allele 2	Bw6	Allele 1	Allele 2	haplotype	*L1*	*L2*	*L3*	*L4*	*L5*	*P1*	*S1*	*S2*	*S3*	*S4*	*S5*	*L1*	*L2*	*L3*	*P1*	*S1*	haplotype
BC170705A	A*01:01	A*11:01	B*08:01	B*51:01	Bw4	C*07:01	C*15:02	C1/C2	1	0	1	1	1	1	1	0	0	0	1	1	1	1	1	1	B
BC170705B	A*02:01	A*26:01	B*07:02	B*51:07	Bw4	C*07:02	C*14:02	C1/C1	1	1	1	1	0	1	0	1	0	1	0	1	1	1	1	0	B
BC170816A	A*24:02	A*31:01	B*13:02	B*15:01	Bw4	C*03:03	C*06:02	C1/C2	1	0	1	1	0	1	0	0	0	0	0	1	1	1	1	0	A
BC170816B	A*02:01	A*23:01	B*15:01	B*44:03	Bw4	C*03:04	C*04:01	C1/C2	1	0	1	1	0	1	0	0	0	0	0	1	1	1	1	0	A
BC170830A	A*01:01	A*24:02	B*27:05	B*44:02	Bw4	C*02:02	C*05:01	C2/C2	0	1	0	1	1	0	1	1	0	0	1	1	1	1	1	0	B
BC170830B	A*02:01	A*11:01	B*07:02	B*35:01	Bw6	C*04:01	C*07:02	C1/C2	1	0	1	1	0	1	0	0	0	0	0	1	1	1	1	0	A
BC171023A	A*23:01	A*33:03	B*35:08	B*44:03	Bw4	C*04:01	C*04:01	C2/C2	1	0	1	1	1	1	1	0	0	0	1	1	1	1	1	1	B
BC171023B	A*02:01	A*02:01	B*15:01	B*44:02	Bw4	C*03:04	C*05:01	C1/C2	1	1	1	1	1	1	0	1	1	0	0	1	1	1	1	0	B
BC171023C	A*02:01	A*02:01	B*15:01	B*51:01	Bw4	C*03:04	C*14:02	C1/C1	1	1	1	1	1	1	1	1	1	0	1	1	1	1	1	1	B
BC171023D	A*03:01	A*26:15	B*07:02	B*38:01	Bw4	C*07:02	C*12:03	C1/C1	1	1	1	1	0	1	0	1	0	1	0	1	1	1	1	0	B
BC171101A	A*01:01	A*02:01	B*08:01	B*57:01	Bw4	C*07:01	C*07:01	C1/C1	1	1	1	1	1	1	1	1	1	0	0	1	1	1	1	1	B
BC171101B	A*02:01	A*24:02	B*13:02	B*15:01	Bw4	C*03:03	C*06:02	C1/C2	1	0	1	1	0	1	0	0	0	0	0	1	1	1	1	0	A
BC171101C	A*02:01	A*24:02	B*07:02	B*44:02	Bw4	C*05:01	C*07:02	C1/C2	1	1	1	1	0	1	0	1	0	1	0	1	1	1	1	0	B
BC171101D	A*03:01	A*26:15	B*07:02	B*38:01	Bw4	C*07:02	C*12:03	C1/C1	1	1	1	1	0	1	0	1	0	1	0	1	1	1	1	0	B
BC171213A	A*01:01	A*31:01	B*44:02	B*57:01	Bw4	C*05:01	C*06:02	C2/C2	1	1	0	1	1	1	1	1	1	0	0	1	1	1	1	1	B
BC171213B	A*01:01	A*23:01	B*08:01	B*45:01	Bw6	C*07:01	C*16:01	C1/C1	1	0	1	1	1	1	1	0	1	0	0	1	1	1	1	1	B
BC171213C	A*02:01	A*03:01	B*07:02	B*15:01	Bw6	C*03:04	C*07:02	C1/C1	1	0	1	1	1	1	1	0	0	0	1	1	1	1	1	1	B
BC171213D	A*03:01	A*30:01	B*13:02	B*51:01	Bw4	C*06:02	C*14:02	C1/C2	0	1	0	1	0	0	0	1	0	1	0	1	1	1	1	0	B

1 indicates the presence of the respective KIR gene, 0 indicates the absence of the respective KIR gene.
